# Hepatitis B Virus Infection Does Not Significantly Influence *Plasmodium* Parasite Density in Asymptomatic Infections in Ghanaian Transfusion Recipients

**DOI:** 10.1371/journal.pone.0049967

**Published:** 2012-11-21

**Authors:** Graham Lee Freimanis, Shirley Owusu-Ofori, Jean-Pierre Allain

**Affiliations:** 1 Division of Transfusion Medicine, Department of Haematology, University of Cambridge, Cambridge, United Kingdom; 2 Transfusion Medicine Unit, Komfo Anokye Teaching Hospital, Kumasi, Ghana; University of Cincinnati College of Medicine, United States of America

## Abstract

**Background:**

Areas endemic for malaria and Hepatitis B virus (HBV) infection largely overlap geographically. A recent study has suggested the existence of an interaction between the two pathogens in symptomatic co-infected individuals on the South-American continent. We examined this issue in a hyperendemic area for both pathogens in sub-Saharan Africa.

**Methodology and Findings:**

Pre-transfusion samples from a retrospective cohort of 154 blood transfusion recipients were screened for both serological and molecular markers of HBV and *Plasmodium* genomes using species-specific nested PCR and quantitative real-time PCR. Thirty-seven individuals met exclusion criteria and were subsequently eliminated from further analysis. Of 117 participants, 90% of recipients exhibited evidence of exposure to HBV, 42% with HBsAg and/or HBV DNA and 48% anti-HBc reactive without detectable HBV DNA. *Plasmodium* genome prevalence by NAT was 50%. Parasitemic individuals were significantly younger than non-parasitemic individuals (*P* = 0.04). Parasitemia level was not significantly lower in individuals with HBV DNA positive infections compared to those with HBV DNA negative exposures. HBV DNA load was not significantly different in parasitemic and non-parasitemic individuals.

**Conclusion:**

The data presented suggests that, in sub-Saharan Africa, asymptomatic co-infections with these two ubiquitous pathogens do not appear to significantly affect each other and evolve independently.

## Introduction

Despite being the focus of extensive research in recent years malaria remains a significant cause of morbidity worldwide, with 1 million deaths a year in sub-Saharan Africa (SSA) alone [1]. In addition to this burden, SSA also has a high prevalence of other clinically significant pathogens, including Hepatitis B virus (HBV) and Human Immunodeficiency virus (HIV) [2]–[4]. Consequently, there are numerous areas of SSA where the endemicity of both malaria and HBV overlap [5]–[7]. Furthermore, with both infections sharing an intra-hepatic stage in their life cycles, interactions between the two pathogens have been hypothesized to occur at both immunological and cellular levels. Such interactions have already been reported in mice [8]. Intriguingly, both pathogens may also utilize common receptors during the hepatocyte invasion [9], [10].

Despite these findings, studies of *Plasmodium* and HBV co-infection are few and there is no clear consensus whether the clinical status of HBV impacts upon *Plasmodium* infection, or vice versa [11], [12]. A previous study examining HBV and *Plasmodium* co-infections suggested that increased viremia in individuals with severe malaria was likely due to decreased HLA expression [13]. Furthermore, lower circulating parasite density in individuals asymptomatically co-infected with both HBV and *Plasmodium*, than in HBV naive individuals suggested a cross-reactive immune response affecting both pathogens [14]. Another study conducted in Gabon however, found no significant correlation between the two pathogens [15]. Neither account identified the HBV genotypes involved nor the impact this had on the results, despite the likelihood of different genotypes being present on different continents [16]. Taking into account the significant serological overlap and the high prevalence of both virus and parasite in Ghana (20% and 50% respectively) [4], [17], a population of hospitalized adult patients asymptomatic for both infections was studied.

## Methods

### Samples

Whole blood samples were collected pre-transfusion from a cohort of 154 Ghanaian transfusion recipients hospitalized in the departments of Medicine and Obstetrics and Gynaecology, at the Komfo Anokye Teaching Hospital (KATH) in Kumasi, Ghana as part of the Blood & Organ Transmitted Infectious Agents (BOTIA) sample repository [18]. All 154 samples were selected at random from the repository using an online tool (http://www.randomizer.org/) to avoid selectional bias. Female recipients (N = 130; average age: 31.9 years) were mostly pregnant (N = 87), hospitalized for massive bleeding related to ectopic pregnancy (N = 16), post-partum hemorrhage (N = 10), abortion (N = 15), or other causes of anemia (N = 46). Non-pregnant women presented with hematological anemia (N = 5), gastro-Intestinal (GI) bleeding (N = 3) or other conditions including diabetes, polyps, fibroids and trauma (N = 35). Male recipients (N = 24; average age: 37.5 years) presented with hematological anemia (N = 3), GI bleeding (N = 7) or severe anemia (N = 10). Other conditions included renal failure and pneumonia (N = 4).

EDTA-treated plasma and cellular fractions were separated and frozen at ≤−40°C until tested as described previously [19] . After initial screening, 37 individuals were excluded from further analysis. Exclusion of these samples was based upon positivity with at least one of the following exclusion criteria: anti-HIV-1/2 (N = 11), anti-HCV (N = 5), receiving anti-malarial therapy (N = 13), diagnosed with sickle cell anemia (N = 13) and Glucose-6 Phosphate Dehydrogenase deficiency (G6PD) (N = 3).

### Ethics Statement

Approval for the BOTIA repository and its use was obtained from the Kwame Nkrumah University of Science and Technology School of Medical Sciences committees for ethics and publication (Kumasi, Ghana). The BOTIA scientific committee approved the present study. Written informed consent was obtained from all participants prior to enrollment.

### Study Locality

Kumasi, located in the central Ashanti region, is the second largest city in Ghana with a population of 1.63 million [20]. The climate is semi-humid tropical and malaria transmission is intense and perennial with Annual Entomological Inoculation Rates (AEIRs) of neighboring areas of Kona and Afamanaso (both within the Ashanti region) being 490 and 866 infectious bites per year respectively [21].

### Serological Testing

All plasma fractions were tested for serological markers to HIV and HCV using the HIV-1/2 DETERMINE rapid tests (Abbott, Maidenhead, UK) and Murex Anti-HCV (version 4) (DiaSorin, Dartford, UK) respectively. Samples reactive for anti-HIV-1/2 were confirmed with the Murex HIV-1.2.0 EIA (DiaSorin) and anti-HCV reactive samples were confirmed using the MonoLisa Anti-HCV-PLUS (version 2) (Bio-Rad). Upon exclusion of 37 individuals meeting study exclusion criteria, 117 participants were screened for HBsAg using the One-step HBsAg rapid test (01FK11) (Standard Diagnostics, Kyonggi-do, S. Korea). Samples non-reactive with the rapid test were tested with the Murex HBsAg enzyme immunoassay (EIA) (DiaSorin). Confirmed HBsAg non-reactive samples were further tested for antibodies against Hepatitis B core antigen (anti-HBc) with the Monolisa Anti-HBc EIA (Bio-Rad, Hemel Hempstead, UK) to confirm previous viral exposure.

### HBV DNA Detection

Viral DNA was extracted from 200 µl of plasma, taken from each of the 117 blood recipient’s using the QIAamp DNA minikit (Qiagen, Crawley, UK) according to manufacturers instructions. HBV DNA was tested by using a real-time qPCR assay targeting HBV S-gene [4] and confirmed with a hemi-nested PCR within the basic core promoter/pre-core region (BCP/PC) [22] and/or a second nested PCR amplifying a 1,434 bp amplicon encompassing the entire pre-S/S gene [23]. In 6 HBsAg positive/HBV DNA unconfirmed samples a third nested-PCR was used to amplify a 276 bp fragment of the S gene [4]. The limit of detection (LOD) of the HBV qPCR assay was 10 IU/ml. The LODs for the hemi-nested assays were, 50 IU/ml for the BCP and S-specific assays and 100 IU/ml for the pre-S/S PCR assay. Sequences of BCP, Pre-S/S and S PCR amplicons were obtained by direct sequencing of PCR products. Amplified products were purified from agarose gel excised bands using Wizard gel and PCR purification kits (Promega, Wallisellen, Switzerland). Ghanaian sequences were aligned with reference HBV genotypes A–H sequences using the CLUSTAL W software implemented within Mac Vector version 10.0.2 software (MacVector). Phylogenetic analysis was performed using the PAUP 4.01b10 software. To confirm the reliability of phylogenetic trees, bootstrap re-sampling was performed for each analysis (1000 replicates). Samples negative by nucleic acid testing were further tested with a real-time PCR targeting the Human Apoprotein B (HAPB) gene as described previously [24] to exclude the presence of potential amplification inhibitors.

### Plasmodium DNA Detection with Species-specific Nested PCRs

DNA was extracted from 200 µl red cell fractions using the QIAamp blood minikit (Qiagen) as per manufacturer’s instructions. All samples were tested twice, in duplicate using a genus-specific primer pair and four species-specific primer pairs (targeting the 18 s ribosomal DNA sequence of *P.falciparum, P.vivax, P.malariae and P.ovale*) in a nested PCR, as described previously [25], [26]. All test runs required validation from positive and negative controls and all amplicons were sequenced to confirm species identity and exclude contamination. Sequencing was carried out at the Department of Biochemistry Sequencing Service, University of Cambridge (http://www.bio.cam.ac.uk/~pflgroup/DNA_Facility/). Samples’ negative for nucleic acid testing were retested with the HAPB real-time PCR assay as described above.

### Pan-*Plasmodium* Real-time Quantitative PCR (qPCR)

All samples positive by nested PCR were retested using a real-time PCR assay targeting the 18 s ribosomal DNA sequence of Plasmodiae [27]. Assays were carried out using the Brilliant Core real-time PCR reagents (Agilent, La Jolla, CA, USA) on an MX3005 thermocycler (Agilent) in a total volume of 25 µl, containing 5 µl of DNA, 250 nM of each primer and 50 nM of probe. Cycling conditions were: 95°C for 10 minutes, followed by 40 cycles of 95°C for 15 s and 60°C for 1 minute. The reference standard was derived from a culture of 3D7 quantified by microscopy and serially diluted prior to DNA extraction. The limit of detection for all 4 *Plasmodium* species was 2 copies/µl. All samples were tested in duplicate on 2 separate runs, with each test run requiring validation by positive/negative controls and the standard curve. For quality control purposes, every sample run included a minimum of two previously quantified samples.

### Statistical Analysis

Analysis was carried out using the GraphPad Prism software 4.0. Continuous variables were compared using the non-parametric Mann-Whitney test. All values shown were derived from the results of a two-tailed test. Nonparametric correlation between groups was calculated using the Spearman test. Multiple group sample comparison was performed using the Kruskal-Wallis test with Dunn’s multiple comparisons. *P*<0.05 was considered statistically significant.

## Results

### HBV Prevalence

Pre-transfusion samples were collected from 154 blood recipients attending KATH in Kumasi, Ghana and tested for serological and molecular markers to determine HBV infection status. Thirty-seven samples were excluded from further analysis as they fulfilled exclusion criteria (see Materials and Methods).

Of the 117 participants remaining in the study (Table 1), 22 (18.8%) were reactive for HBsAg by rapid-test. All 22 samples were further characterized as HBsAg+/HBV DNA+ (median viral load: 2.1e2 IU/ml; range 3.8×10e0–4.9×10e6 IU/ml). The 95 samples non-reactive for HBsAg by rapid test were re-tested by HBsAg EIA that identified 20 (21%) as positive (median S/CO: 1.6; median viral load 1.0×10e3 IU/ml; range 2.0×10e2–1.0×10e4 IU/ml). Overall, 42 (36%) recipients were HBsAg positive by either rapid test or EIA (median age: 28.5 years) with 41 (98%) positive by NAT with HBV DNA load ranging between 1.45×10e+1 and 4.9×10e+6 IU/ml (Table 2). All 42 were anti-HBc reactive, despite one sample (0.8%) being HBsAg+/HBV DNA(-).

**Table 1:** Age, gender and HBV status of a population of 117 pre-transfusion recipient patients.

Gender			
	Male (%)	Female (%)	All
N	14	103	117
Average age (years)	40.5	30	30
**Age group (years)**			
	**Male (%)**	**Female (%)**	**N (%)**
<20	1 (7.1)	8 (7.8)	9 (7.6)
20–29	4(28.6)	40 (38.8)	44 (37.6)
30–39	1 (7.1)	35 (34)	36 (30.8)
40–49	3 (21.4)	10 (9.7)	13 (11.1)
≥50	5 (35.8)	9 (8.7)	14 (12)
Unknown	–	1 (1)	1 (0.9)

Of 75 HBsAg negative patient samples, 63 tested anti-HBc reactive. Of these, 56 (48%) samples were identified as ‘HBV recovered’ (HBsAg−/anti-HBc+/HBV DNA(-)), whilst 7 (6%) exhibited detectable levels of HBV DNA indicating occult HBV infection (OBI). The remaining 12 recipients (10%) were characterized as HBsAg−/anti-HBc−/HBV DNA(-) and considered ‘HBV susceptible’ (Table 2). All 69 samples identified as HBV DNA negative by NAT, tested positive for Human Apoprotein B gene (HAPB) DNA (confirming their negative status).

**Table 2:** HBV and Plasmodium screening in pre-transfusion blood recipient samples.

Total tested		154		
		Parasitemic	Non-parasitemic	
Exclusion criteria* §	HIV+ : Confirmed Anti-HIV+(%)	11 (7.1)		
	: HIV-Plasmodium co-infection	8		3
	: Median HBV Viral load (IU/ml)	3.4e2		1.00e(-)1
	: Median Parasitemia (parasites/ml)	2.75e+04		
	HCV+: Confirmed Anti-HCV+(%)	5 (3.2)		
	: HCV-Plasmodium co-infection	3		2
	: Median HBV Viral load (IU/ml)	1.00e(-)01		1.00e(-)1
	: Median Parasitemia (parasites/ml)	2.9e+05		
	Received antimalarial therapy (%)	13 (8.4)		
	Sickle cell anemia (%)	13 (8.4)		
	Glucose-6 Phosphate Dehydrogenase deficiency (%)	3 (1.9)		
**Total included in analysis**		117		
*Plasmodium*	Total parasitemic/Non-parasitemic (%)	58 (49.6)		59 (50.4)
	Single infection (Pf)	52 (89.7)		–
	Mixed infection (Pf/Pm)	5 (8.6)		–
	Mixed infection (Pf/Po)	1 (1.7)		–
	Median Parasitemia (parasites/ml)	8.37e+02		–
HBV	HBV infection [HBsAg+/HBV DNA+](%)	42 (35.9)		
	Parasitemic status	25		17
	Median Viral load (IU/ml)	1.0e+3		4.61e+2
	Median Parasitemia (parasites/ml)	4.31e+2		–
	Occult HBV infection [HBsAg−/anti-HBc +/HBV DNA+](%)	7 (5.9)		
	Parasitemic status	4		3
	Median Viral load (IU/ml)	7.75e+01		2.0e+2
	Median Parasitemia (parasites/ml)	1.95e+3		–
	Recovered HBV [HBsAg−/anti-HBc+/HBV DNA−] (%)	56 (47.9)		
	Parasitemic status	24		32
	Median Parasitemia (parasites/ml)	3.44e+3		–
	HBV Susceptible [HBsAg−/Anti-HBc−/HBV DNA(-)] (%)	12 (10.3)		
	Parasitemic status	5		7
	Median Parasitemia (parasites/ml)	1.63e+3		–

* 8 individuals were positive for >1 exclusion criteria.

§ 37 individuals were excluded from further analysis when investigating associations between HBV and *Plasmodium* parasitemia.

The 48 DNA positive samples were sequenced in the pre-S/S, S or BCP-PC regions and genotyped by phylogenetic analysis using a panel of genotyped samples from Genbank. All sequences clustered with genotype E (data not shown). All sequences have been submitted to Genbank under accession numbers JX982150–JX982218.

### *Plasmodium* DNA Prevalence

DNA extracted from the 117 patient cellular fractions was tested for evidence of parasitemia (Table 2). Nested PCR identified 58 (49.6%) pre-transfusion samples with detectable *Plasmodium* genome. Of these, 52 (90%) carried single species P.*falciparum* infections; five (9%) carried mixed infections of P.*falciparum/*P.*malariae* and one (2%) exhibited a mixed infection of P.*falciparum*/P.*ovale* (Table 2). Quantitative PCR results were concordant with nested PCR in 55 samples (95%), with the *Plasmodium* identity of each amplicon confirmed by sequencing. The median level of parasitemia was 8.4×10e+2 parasites/ml. Fifty-nine samples negative for *Plasmodium* DNA by NAT were retested with the HAPB real-time PCR and were found positive.

### Correlation between HBV Exposure and *Plasmodium* Parasitemia

**Figure 1:**
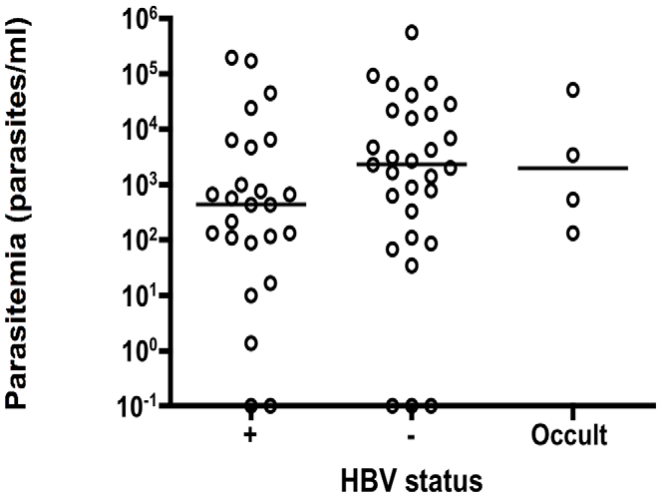
Plasmodium parasitemia in asymptomatic transfusion recipient samples stratified according to HBV status. Horizontal bars indicate median level of parasitemia. Samples on the bottom 10^−1^ line indicate a positive signal too weak to allow quantification.

In order to study associations between HBV and *Plasmodium*, parasite density was stratified according to HBV status in the 117 samples (Figure 1). In total, 49 samples (42%) were positive for HBV (HBsAg and/or DNA) with 7 of these identified as occult HBV infections (HBsAg−/DNA+). Of these, 25/42 HBV positive and 4/7 OBI infected individuals exhibited parasitemia. These were compared to 29 HBV−/Plasmodium+ individuals, from a total of 68 (43%) HBV negative participants (Figure 1). The difference between parasitemia levels in HBV infected (HBsAg+/DNA+), HBV OBI and HBV negative (HBsAg−/DNA(-)) samples was not significant (*P* = 0.09, Kruskal Wallis test) although the HBV negative group exhibited a median level of parasitemia nearly one log above the active infection group.

**Figure 2:**
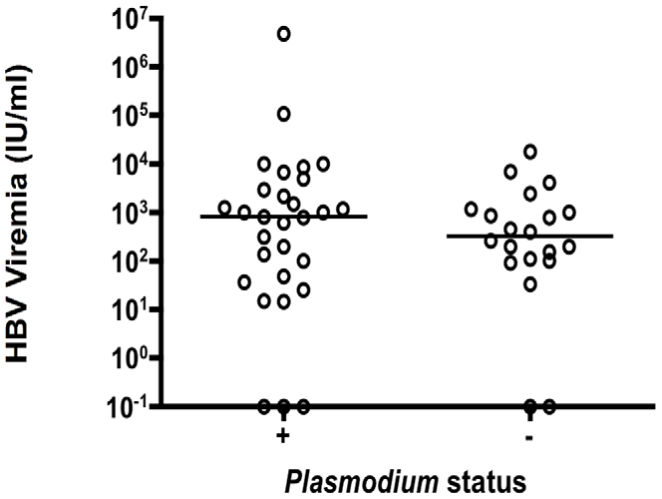
HBV DNA load stratified according to parasitemic status in asymptomatic transfusion recipients. Horizontal bar indicate median level of viral load. Samples on the 10e(-)1 line correspond to positive HBV DNA signal too weak to allow quantification.

HBV DNA viral load was stratified according to parasitemic status (Figure 2). Among 58 parasitemic individuals, 29 (50%) were HBV infected (HBsAg/DNA) with viral loads compared to that in 20 HBV+/Plasmodium negative individuals. Median HBV viral load was increased in parasitemic individuals, compared to non-parasitemic but the difference between the two groups was not significant (Mann-Whitney, *P* = 0.5).

**Figure 3:**
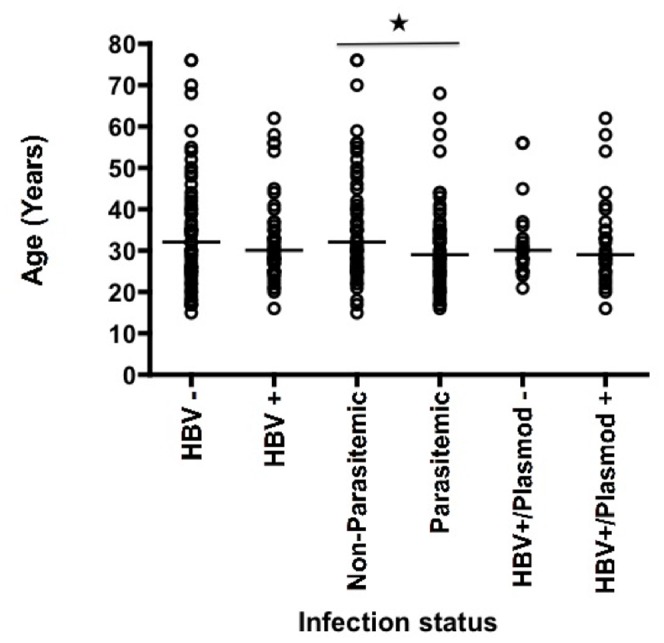
Age distribution of Ghanaian transfusion recipients stratified according to HBV and malaria parasitemia status. Number of samples included = 117. HBV negative (N = 68); HBV positive (N = 49); Plasmodium negative (N = 59); Plasmodium positive (N = 58); HBV positive/Plasmodium negative (N = 20); HBV positive/Plasmodium positive (N = 29). *: Age distribution of parasitemic and non-parasitemic patients (Mann-Whitney, P = 0.0397).

The effect of age on infection status was examined (Figure 3). The age distribution of patients with active or recovered HBV infection or naïve was not significant (median age: 29 and 30 years respectively). Individuals who were parasitemic were significantly younger than those who were non-parasitemic (median ages: 32 and 27.5 years respectively) (Mann-Whitney, *P* = 0.04). The age distribution of parasitemic or non-parasitemic recipients with active HBV infections (median age: 30 and 28 years respectively) was not significantly different (Mann-Whitney, *P* = 0.081).

The potential influence of HIV infection upon parasitemia was examined by comparing parasitemia levels of 8/11 parasitemic HIV positive individuals and 53 parasitemic/HIV negative individuals. No significant difference was found (Mann-Whitney, *P* = 0.34).

## Discussion

The aim of this study was to verify the potential interactions, as suggested by previous studies, between HBV (viremia) and *Plasmodium* parasite density in asymptomatic co-infected and single infected patients hospitalized at Komfo Anokye Teaching Hospital, Kumasi, Ghana. Both pathogens commonly exhibit overlapping regions of endemicity, particularly in sub-Saharan Africa and have a significant clinical impact upon individuals residing in these regions. In Kumasi, Ghana, it has been shown previously that by the age of 40, 100% of the blood donor population has been in contact with HBV, with 15–20% carrying detectable viral genome [4]. Recent work in our laboratory has also indicated that 100% of the adult population was semi-immune to *Plasmodium* with over 50% carrying detectable parasite DNA in the blood [17]. As a result it could be predicted that approximately 10% of the adult population harbored co-circulating detectable HBV and *Plasmodium* DNA and was therefore highly suitable to investigate potential interaction between the two pathogens circulating in a sub-Saharan African asymptomatic adult population.

Although previous studies have addressed potential interactions between HBV and *Plasmodium*, the majority has been limited by small samples numbers [11] or focused specifically on clinical malaria [13]. A recent study in South-America identified significantly lower parasite density in asymptomatic individuals with active HBV infections, than those without HBV infections [14]. Additionally, this study confirmed observations of significantly increased HBV viral load in those who were parasitemic, compared to HBV only infections as reported previously in Gambian children with severe malaria [13]. In both instances the differences observed in our investigation were not significant, supporting another previous study [28]. The observations presented here should be considered taking several key factors into account.

Firstly, the HBV genotypes involved in both studies are likely to differ. The dominant HBV genotypes circulating in Brazil are A, D and F [29], [30], whereas the predominant HBV genotype reported in Ghana is E [31]. Furthermore, differences in HBV prevalence [22], [30] and vaccination coverage [32], [33] are also likely to influence subsequent associations. Important differences also exist in the molecular epidemiology of the *Plasmodium* parasites, at different study sites. P.*vivax* causes the majority of *Plasmodium* infections on the South-America continent (84%) with the minority due to P.*falciparum* (16%) [16]. Furthermore, levels of parasite prevalence in the Brazilian Amazon region are heterogeneous with a significant proportion of asymptomatic infections within specific communities [34]. In Ghana, the overall prevalence of parasitemia in asymptomatic adults exceeds 50% [35] with P.*falciparum* accounting for >90% of cases [17]. Furthermore, Ghanaian patients exhibited a prevalence of mixed species including P.*falciparum*/P.*malariae* and P.*falciparum*/P.*ovale*, with at least P.*ovale* not being present within South American parasite populations [16]. A recent study investigating HBV and *Plasmodium* co-infections in sub-Saharan Africa in an area with a similar prevalence of P.*falciparum* (>80%) failed to demonstrate an association between HBV and *Plasmodium* infections, although a significant link with HCV was identified. Reduced differences observed between experimental groups may also reflect the asymptomatic status of patients included within the study, as observed previously [14]. With one or both infections contained by the host immune system and in the absence of clinical pathology, this data may also suggest that there are no significant interactions between the two pathogens.

The data presented in this study of 117 hospitalized patients asymptomatic for both HBV and malaria confirmed HBV epidemiologic predictions, since 90.3% had been exposed to HBV although 42.2% instead of the predicted 20% of patients had active HBV infection defined by the presence of viral DNA in plasma. The difference in prevalence of viremia might be in part related to data from blood donors being collected in a younger population, a fifth of whom had previously tested negative for HBsAg (repeat donors). It may also be related in part to the use of a more sensitive assay for HBV DNA detection reflected in the prevalence of HBsAg negative/HBV DNA positive occult HBV infections found at 1.6% in blood donors but 7.1% in patients. In a population of pregnant women in Kumasi, 16% had active infections but only 1.5% were occult [23]. In this latter study, 5.4% of HBsAg positive samples were HBV DNA negative while in the present study only 2.3% were DNA negative, strongly suggesting an increased sensitivity of HBV DNA detection. In addition, the phylogenetic analysis indicated that all sequenced samples were genotype E, confirming previous reports [23], [36].

The prevalence and molecular epidemiology of *Plasmodium* presented here also supported previous reports (50% and 55%, respectively) [35] with the majority of parasitemic recipients presenting single species P.*falciparum* infection. Data also confirmed that parasitemic individuals were significantly younger than non-parasitemic recipients (Figure 3), confirming similar observations reported in the literature [37], [38]. This is likely to reflect the slow development of semi-immunity efficacy, typically observed in older individuals who reside in areas with high transmission intensity [39].

Whilst steps have been taken to minimize the impact of host genetic and external factors i.e. other pathogens, these efforts were not exhaustive. Despite the heterogeneous nature of clinical complications suffered by patients included in this study, none presented clinical conditions relating to hepatitis or liver dysfunction. Furthermore, the impact of other pathogens capable of modulating *Plasmodium* infections was also considered, particularly with respect to Helminthes infections, although given their low prevalence within Ghanaian urban environments [40] this impact was not considered significant.

### Conclusions

No conclusive evidence of interaction between HBV and *Plasmodium* was found in cases of co-infections in a holoendemic region. The data presented here suggests that in an area of high endemicity for *Plasmodium falciparum* and HBV genotype E in sub-Saharan Africa, both pathogens appear likely to evolve independently of one another in asymptomatic infections.
